# Benchmarking HIV health care: from individual patient care to health care evaluation. An example from the EuroSIDA study

**DOI:** 10.1186/1471-2334-12-229

**Published:** 2012-09-25

**Authors:** Daria N Podlekareva, Joanne Reekie, Amanda Mocroft, Marcelo Losso, Aza G Rakhmanova, Elzbieta Bakowska, Igor A Karpov, Jeffrey V Lazarus, Jose Gatell, Jens D Lundgren, Ole Kirk

**Affiliations:** 1 Copenhagen HIV Programme, University of Copenhagen, Copenhagen, Denmark; 2 University College London Medical School, Royal Free Campus, London, UK; 3 Hospital JM Ramos Mejia, Buenos Aires, Argentina; 4 Botkin Hospital of Infectious Diseases, St Petersburg, Russia; 5 Wojewodzki Szpital Zakazny, Warszawa, Poland; 6 Belarus State Medical University, Minsk, Belarus; 7 Hospital Clinic de Barcelona, Barcelona, Spain; 8 Department of Infectious Diseases, Rigshospitalet, Copenhagen, Denmark

**Keywords:** HIV-infection, HIV health care, Health care interventions, Health care benchmark, HIV in Eastern Europe, HIV in Europe

## Abstract

**Background:**

State-of-the-art care involving the utilisation of multiple health care interventions is the basis for an optimal long-term clinical prognosis for HIV-patients. We evaluated health care for HIV patients based on four key indicators.

**Methods:**

Four indicators of health care were assessed: Compliance with current guidelines on initiation of: 1) combination antiretroviral therapy (cART); 2) chemoprophylaxis; 3) frequency of laboratory monitoring; and 4) virological response to cART (proportion of patients with HIV-RNA < 500copies/ml for >90% of time on cART).

**Results:**

7097 EuroSIDA patients were included from Northern (n = 923), Southern (n = 1059), West Central (n = 1290) East Central (n = 1366), Eastern (n = 1964) Europe, and Argentina (n = 495). Patients in Eastern Europe with a CD4 < 200cells/mm^3^ were less likely to initiate cART and *Pneumocystis jiroveci*-chemoprophylaxis compared to patients from all other regions, and less frequently had a laboratory assessment of their disease status. The proportion of patients with virological response was highest in Northern, 89% vs. 84%, 78%, 78%, 61%, 55% in West Central, Southern, East Central Europe, Argentina and Eastern Europe, respectively (p < 0.0001). Compared to Northern, patients from other regions had significantly lower odds of virological response; the difference was most pronounced for Eastern Europe and Argentina (adjusted OR 0.16 [95%CI 0.11-0.23, p < 0.0001]; 0.20[0.14-0.28, p < 0.0001] respectively).

**Conclusions:**

This assessment of HIV health care utilization revealed pronounced regional differences in adherence to guidelines and can help to identify gaps and direct target interventions. It may serve as a tool for the assessment and benchmarking of the clinical management of HIV patients in any setting worldwide.

## Background

Standards of care for HIV patients are established by national and international guidelines, but vary across the world depending on the economic situation in a particular country, health system infrastructures and the prevalence of HIV [1-4]. State-of-the-art care of HIV patients requires the utilisation of multiple health care interventions, not only limited to laboratory and clinical procedures for disease monitoring, but also involving health system multisectoral interventions such as the procurement of antiretroviral drugs and laboratory equipment. In many countries, access to combination antiretroviral therapy (cART) is limited as are choice of drugs and treatment options, and monitoring of treatment efficacy with CD4-cell count and HIV-RNA measurements is often not possible [5].

Proper utilisation of health care interventions depends on several factors including health system financing and prioritisation, qualification and knowledge level of physicians, as well as the patients’ ability to access the relevant health care system at the most appropriate time. Deficits in provided care may have important implications for the health of the individual HIV patient as well as for public health [6]. Several attempts have been made to develop HIV care quality measures which are typically limited to one country or a single clinic [7,8]. The World Health Organization (WHO) has also developed a set of indicators for monitoring the health systems response to HIV/AIDS [9], which are mainly focused on health systems per se without the capability to monitor clinical management of patients (i.e. their immunological and virological status). Currently, there is a lack of knowledge about health care utilization on a multinational level within resource-rich and -poor settings, in particular health care adherence to the clinical guidelines on management of HIV infection. The development of uniform measures for HIV care quality would allow cross-regional comparison and the identification of a benchmark for HIV care.

The EuroSIDA study represents a unique opportunity for assessing the clinical management of HIV infection on an individual level as well as enabling comparison across different regions in Europe and Argentina. Our objectives were to evaluate the performance of four health care indicators (HCI), all based on current HIV treatment guidelines [1-4], and compare their utilisation across regions of Europe and Argentina in order to suggest a clinical benchmark for HIV health care.

## Methods

### EuroSIDA study

EuroSIDA is a prospective observational cohort study of 16,597 HIV patients from 103 clinics in 35 countries (Europe, Israel and Argentina). The study’s details have been described elsewhere [10,11].

This analysis is based on patients recruited into the study after 2001, when EuroSIDA expanded its network to Eastern Europe. Information is collected on a standardized, adjustable data collection form at enrolment and every six months thereafter ( http://www.eurosida.org). Information collected includes all CD4-cell counts and HIV-RNA measured since the last follow-up, dates of starting and stopping each antiretroviral drug as well as drugs used for treatment and chemoprophylaxis against opportunistic infections. Dates of diagnosis of all AIDS-defining illnesses are also recorded using the 1993 clinical definition of AIDS from the US Centers for Disease Control [12]. A comprehensive quality assurance programme has been established to ensure correct patient selection and to verify that accurate data are supplied. The study was approved by the Ethics Committees of participating clinics (N = 103), as per local and national regulations. All patients’ data were obtained from patients’ medical records or via database exchange using HICDEP format ( http://www.hicdep.org). Patients had to sign Informed Consent where it was requested by local regulations.

### Statistical methods

Patients eligible for this analysis were those enrolled in EuroSIDA from 2001 onwards with at least one follow-up visit after baseline. For regional analysis of HCI, six regions were established according to the country of residence of the patient:

• Southern Europe (SE): Greece, Israel, Italy, Portugal, Spain;

• West Central Europe (WCE): Austria, Belgium, France, South Germany, Luxembourg, Switzerland;

• North Europe (NE): Denmark, Finland, North Germany, Ireland, the Netherlands, Norway, Sweden, the United Kingdom;

East Central Europe (ECE): Bulgaria, Croatia, the Czech Republic, Hungary, Poland, Romania, Serbia and Slovakia;

• Eastern Europe (EE): Belarus, Estonia, Latvia, Lithuania, the Russian Federation and the Ukraine;

• Argentina (AR)

**Figure 1:**
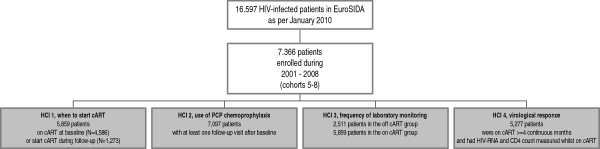
**Inclusion criteria for EuroSIDA patients in the analysis of four different Health Care Interventions (HCI).** cART, combination antiretroviral therapy; baseline = date of enrolment into the EuroSIDA study. Patients for each HCI were selected from 7,366 patients based on the inclusion criteria explained below. HCI 1: all patients who ever started cART (either prior to or after baseline) were included (5859 patients, 4586 on cart at baseline, 1273 start cart after baseline). HCI 2: all EuroSIDA patients, enrolled after 2001 and with a minimum of one follow-up visit (7097). HCI 3: the off cART group included patients who were off cART at baseline and subsequently have not started cART. If patients started cART, they were censored at the time of starting cART. The on cART group included patients who were on cART at baseline or started cART during follow-up (5859 on cart group and 2511 in the off cART group). HCI 4: patients who were on cART for at least 4 consecutive months and had their CD4-cell count and HIV-RNA measured whilst on cART. The first 4 months after each start/change of cART were excluded to ensure that periods when viral suppression would not be expected were not included in the measure of HCI [14]. Follow-up was censored if a patient had not had a HIV-RNA measurement for >6 months and continued from the next available HIV-RNA measurement (5277).

Health care provided to HIV patients in different regions was assessed using four HCI, as presented below, and chosen according to the main recommendations of international guidelines on the management of HIV patients [1-4]. The selection of patients for analysis of each HCI is explained in Figure 1.

1. Compliance with current guidelines on when to start cART, based on CD4-cell count or presence of AIDS diagnosis [1;2;3]

2. Compliance with current guidelines on prophylaxis of opportunistic infections, i.e. initiating of *Pneumocystis jiroveci* pneumonia (PCP) chemoprophylaxis at CD4-cell count <200 cells/mm^3^ and no prior diagnosis of PCP [13]

3. Laboratory evaluation of HIV-disease status: median number of CD4-cell count and HIV-RNA measurements performed per patient per follow-up year, stratified by whether patients were off or on cART

4. Virologic response to cART, assessed by the proportion of patients spending more than 90% of the follow-up time on cART with suppressed HIV-RNA (<500 copies/ml) [14].

cART was defined as at least two nucleos(t)ide reverse transcriptase inhibitors (NRTI) plus either one non-nucleoside reverse transcriptase inhibitor (NNRTI), protease inhibitor (PI)/ritonavir boosted (b) PI or abacavir.

Baseline was defined as the date of enrolment into the EuroSIDA study. Descriptive statistics were used to compare patients’ baseline characteristics across the six regions. Baseline CD4-cell counts and HIV-RNA measurements were assessed using measurements closest to, and no longer than, six months prior to baseline.

Logistic regression was used to identify factors associated with suppressed HIV-RNA for more than 90% of the follow-up time on cART. Follow-up after starting cART was limited to the actual time on cART, excluding treatment interruptions and periods of non-cART use. The first 4 months after each start or change of cART were excluded from the analysis to ensure that periods when viral suppression would not be expected were not included in the measure of HCI [14]. Follow-up was censored if a patient had not had a HIV-RNA measurement for >6 months and continued from the next available HIV-RNA measurement. The following variables were considered in univariable analyses: region of residence, gender, age, race, risk factors for HIV exposure, type of cART regimen, treatment naïve at starting cART, hepatitis B/C (HBV/HCV) status, date of starting cART, baseline and nadir CD4-cell count, baseline and maximal HIV-RNA. Factors that were significant in the univariable model (p < 0.1) were then incorporated in the multivariable model. Missing values were included in the analysis as a separate category for the categorical variables; for the continuous variables the inclusion criteria for the time on cART with a suppressed HIV-RNA analysis was having CD4-cell count and HIV-RNA data available.

A sensitivity analysis was performed on a subset of patients on cART with HIV-RNA measurements performed using assays with a 50 copies/ml limit of quantification.

Follow-up until a median last visit date of April 2011 (IQR February 2010-September 2011) was included in the present analysis, and all analyses were performed using SAS (Statistical analysis software, Cary, NC, USA) version 9.1.

## Results

A total of 7366 patients were recruited to EuroSIDA after 2001 and of those 7097 had at least one follow-up visit and were included in this analysis (Figure 1). Patients’ characteristics are shown in Table 1. Patients from EE differed considerably from patients in other regions. They were younger, with a higher proportion of females; half of them were infected with HIV by injecting drug use (IDU) and were coinfected with HCV. 61% of patients in EE were cART-naïve when recruited to the study. A higher proportion of patients from AR than the other regions were infected with HIV by heterosexual contact and more had been diagnosed with AIDS at baseline.

**Table 1:** **Baseline characteristics of the EuroSIDA patients, included in the study after January 1**^**st**^**, 2001 and according to the region of residence**

		**SE**	**WCE**	**NE**	**ECE**	**EE**	**AR**	***p***
		**1059**	**1290**	**923**	**1366**	**1964**	**495**	
**N (% of total)**								
Gender	Male	799 (75.5)	992 (76.9)	702 (76.1)	968 (70.9)	1103 (56.2)	307 (62.0)	<.0001
Ethnic origin	White	952 (90.0)	911 (76.7)	749 (81.9)	1358 (99.6)	1958 (99.9)	488 (98.6)	<.0001
Exposure Group	Homosexual	7426 (40.2)	566 (43.9)	473 (51.3)	508 (37.2)	149 (7.6)	126 (25.5)	<.0001
	IDU	188 (17.8)	128 (9.9)	86 (9.9)	345 (25.3)	923 (47.0)	62 (12.5)	
	Heterosexual	359 (33.9)	404 (31.3)	290 (31.4)	410 (30.0)	791 (40.3)	291 (58.8)	
Treatment History	Naïve	253 (23.9)	183 (14.2)	145 (15.7)	259 (19.0)	1200 (61.1)	82 (16.6)	<.0001
	cART	763 (72.0)	1035 (80.2)	744 (80.6)	1016 (74.4)	632 (32.2)	396 (80.0)	
Hepatitis B surface antigen status	positive	45 (4.3)	58 (4.5)	35 (3.8)	60 (4.4)	107 (5.5)	26 (5.3)	.003
	negative	827 (78.1)	996 (77.2)	742 (80.4)	1115 (81.6)	1562 (79.5)	357 (72.1)	
	unknown	187 (17.7)	236 (18.3)	146 (15.8)	191 (14.0)	295 (15.0)	112 (22.6)	
Hepatitis C antibody status	positive	174 (16.4)	155 (12.0)	81 (8.8)	352 (25.8)	950 (48.4)	91 (18.4)	<.0001
	negative	688 (65.0)	874 (67.8)	662 (71.6)	831 (60.8)	666 (33.9)	295 (59.6)	
	unknown	197 (18.6)	261 (20.2)	180 (19.5)	183 (13.4)	348 (17.7)	109 (22.0)	
Previous AIDS diagnosis	yes	180 (17.0)	352 (27.3)	187 (20.3)	312 (19.9)	391 (19.9)	101 (32.5)	<.0001
**Median (IQR)**								
Age		39 (33–46)	42 (36–49)	42 (36–50)	35 (29–42)	30 (26–36)	36 (31–43)	<.0001
CD4-cell count/mm^3 1^		434 (293–636)	447 (305–628)	432 (310–610)	369 (245–524)	415 (267–575)	346 (199–518)	<.0001
HIV-RNA _(log10)_ copies/ml^2^		2.05 (1.70-4.08)	1.70 (1.70-3.33)	1.70 (1.60-3.28)	2.33 (1.70-3.79)	3.48 (2.60-4.58)	2.19 (1.70-4.00)	<.0001
Baseline date		1/06 (11/03-6/06)	1/06 (11/03-8/06)	1/06 (11/03-6/08)	11/05 (1/02-6/08)	1/06 (2/04-6/08)	2/06 (1/03-6/06)	<.0001

Baseline date defined as the date of enrolment into the EuroSIDA study.

IDU, injection drug user; cART, combination antiretroviral therapy; HIV-RNA, viral load;

SE, Southern Europe: Greece, Israel, Italy, Portugal, Spain; WCE, West Central Europe: Austria, Belgium, France, South Germany, Luxembourg, Switzerland; NE, Northern Europe: Denmark, Finland, North Germany, Ireland, the Netherlands, Norway, Sweden, the United Kingdom; ECE, East Central Europe: Bulgaria, Croatia, the Czech Republic, Hungary, Poland, Romania, Serbia and Slovakia; EE, Eastern Europe: Belarus, Estonia, Latvia, Lithuania, the Russian Federation and the Ukraine; AR, Argentina.

^1^ N (%) CD4-cell count measurements were missing for 33 (3.1), 36 (2.8), 26 (2.8), 67 (4.69), 418(21.3) and 104 (21.0) in SE, WCE, NE, ECE, EE and AR respectively.

^2^ N (%) HIV-RNA measurements were missing for: 52 (4.9), 49 (3.8), 21 (2.3), 252 (18.5), 1289 (65.6) and 132 (26.7) in SE, WCE, NE, ECE, EE and AR respectively.

### HCI 1 Compliance with current guidelines on when to start cART

**Figure 2:**
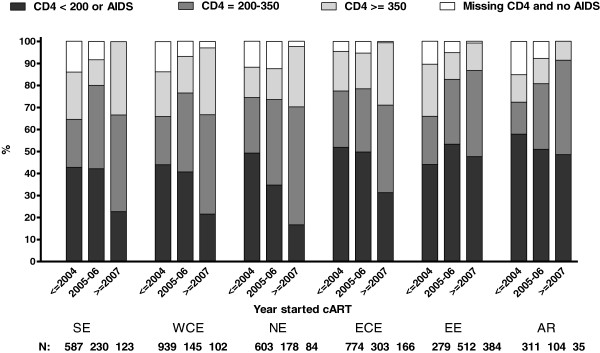
**Proportion of EuroSIDA patients starting cART at various CD4-cell strata and presence of AIDS diagnosis according to the region of residence and calendar year.** SE, Southern Europe: Greece, Israel, Italy, Portugal, Spain; WCE, West Central Europe: Austria, Belgium, France, South Germany, Luxembourg, Switzerland; NE, Northern Europe: Denmark, Finland, North Germany, Ireland, the Netherlands, Norway, Sweden, the United Kingdom; ECE, East Central Europe: Bulgaria, Croatia, the Czech Republic, Hungary, Poland, Romania, Serbia and Slovakia; EE, Eastern Europe: Belarus, Estonia, Latvia, Lithuania, the Russian Federation and the Ukraine.

Figure 2 shows changes over time in the proportions of patients starting cART within different strata of CD4-cell counts according to the region of residence. Of all patients starting cART (N = 5859, Figure 1), the proportion doing so at a CD4-cell count <200 cells/mm^3^ or an AIDS diagnosis decreased by 20% in SE (from 43% to 23%), 22% in WCE (from 44% to 22%), 32% in NE (from 49% to 17%),19% in ECE (from 52% to 31%) and 9% in AR (from 58% to 49%) from ≤ 2004 till ≥ 2007. In EE, however, a larger proportion of patients started cART at CD4-cell count <200 cells/mm^3^ or at AIDS diagnosis in 2007 or later compared with ≤2004 (48% vs. 44%).

Among patients starting cART at CD4-cell count <200 cells/mm^3^, median peak CD4-cell count prior to cART initiation was highest in EE [188 cells/mm^3^ (IQR 106–460 cells/mm^3^)], whereas it was similar in the other regions [from 92 cells/mm^3^ (40–170 cells/mm^3^) in AR till 120 cells/mm^3^ (50–194 cells/mm^3^) in WCE)].

In 2007, HIV treatment guidelines were modified to recommend the initiation of cART at a CD4-cell count ≤350 cells/mm^3^[1-3]. Subsequently, the proportion of patients starting cART at a CD4-cell count of 200–350 cells/mm^3^ increased compared to ≤2004 in SE from 22% to 44%, in WCE from 22% to 45%, in NE from 25% to 54%, in ECE from 26% to 40%, in EE from 22% to 39% and in AR from 14 to 43% (Figure 2).

A total of 1583 patients had been diagnosed with an AIDS defining disease before the baseline. Of those, 216 (14%) did not start cART. The proportion of AIDS-patients who did not start cART was highest in EE, (38%) compared to 4%, 6%, 6%, 8% and 2% from SE, WCE, NE, ECE and AR respectively (p < 0.0001).

### HCI 2 Compliance with current guidelines on prophylaxis of opportunistic infections

**Figure 3:**
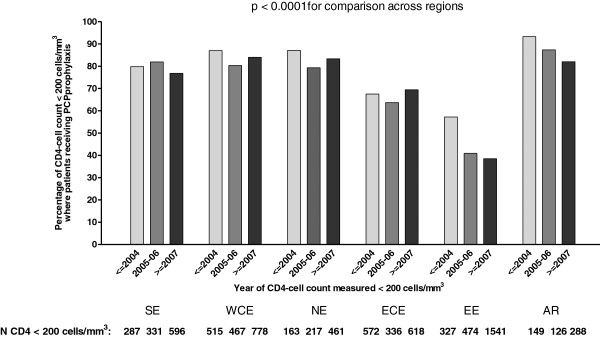
**Proportion of CD4-cell measurements < 200 cells/mm**^**3**^**where patients were receiving prophylaxis for *Pneumocystis jiroveci *pneumonia (PCP) and had not had PCP diagnosis.** SE, Southern Europe: Greece, Israel, Italy, Portugal, Spain; WCE, West Central Europe: Austria, Belgium, France, South Germany, Luxembourg, Switzerland; NE, Northern Europe: Denmark, Finland, North Germany, Ireland, the Netherlands, Norway, Sweden, the United Kingdom; ECE, East Central Europe: Bulgaria, Croatia, the Czech Republic, Hungary, Poland, Romania, Serbia and Slovakia; EE, Eastern Europe: Belarus, Estonia, Latvia, Lithuania, the Russian Federation and the Ukraine; AR, Argentina. cART, combination antiretroviral therapy.

6797 patients were included in this analysis (Figure 1). The proportion of CD4-cell measurements <200 cells/mm^3^ where patients were receiving PCP prophylaxis was generally highest in Argentina in all three time periods (93%, 87% and 83% in < 2004, 2005–2006 and >2007, respectively) and lowest in Eastern Europe (57%, 41%, 38% in < 2004, 2005–2006 and >2007, respectively) (p < 0.0001) (Figure 3).

### HCI 3 Laboratory evaluation of HIV disease status

**Figure 4:**
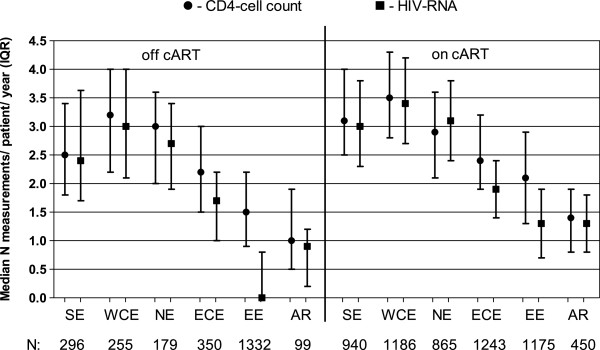
**Frequency of CD4-cell count and HIV-RNA measurements among patients not receiving cART and those receiving cART in the EuroSIDA study.** SE, Southern Europe: Greece, Israel, Italy, Portugal, Spain; WCE, West Central Europe: Austria, Belgium, France, South Germany, Luxembourg, Switzerland; NE, Northern Europe: Denmark, Finland, North Germany, Ireland, the Netherlands, Norway, Sweden, the United Kingdom; ECE, East Central Europe: Bulgaria, Croatia, the Czech Republic, Hungary, Poland, Romania, Serbia and Slovakia; EE, Eastern Europe: Belarus, Estonia, Latvia, Lithuania, the Russian Federation and the Ukraine; AR, Argentina. cART, combination antiretroviral therapy; HIV-RNA, viral load.

There were 2511 and 5859 patients included in the off and on cART analyses, respectively (Figure 1). In general, patients in EE and AR had less frequent CD4-cell count and HIV-RNA measurements per patient per year of follow-up compared with the other regions (Figure 4). There was a median of 0.0 HIV-RNA measurements per patient per year of follow-up in EE (IQR 0.0-0.8) and 0.9 in AR (IQR 0.2-1.2) compared to 1.7 (1.0-2.2) in ECE, 2.7(1.9-3.4) in NE, 3.0 (2.1-4.0) in WCE and 2.4 (1.7-3.3) in SE in the off cART group, p < 0.0001. Even though patients in EE and AR who were on cART had their HIV-RNA measured slightly more often [1.3 (0.7-1.9) and 1.3 (0.8-1.8), respectively], it was still significantly less frequent compared to the other regions, p < 0.0001. Differences in the number of CD4-cell count measurements followed the same pattern (Figure 4).

### HCI 4 Virologic response to cART

There were 5277 patients who were on cART for more than 4 consecutive months with CD4-cell count and HIV-RNA measurements available (Figure 1).

The proportion of patients spending more than 90% of the follow-up time on cART with HIV-RNA < 500 copies/ml was also highest in NE, 89% compared with 84% in WCE, 78% in SE and 78% ECE and only 61% and 55% in AR and EE, p < 0.0001.

**Figure 5:**
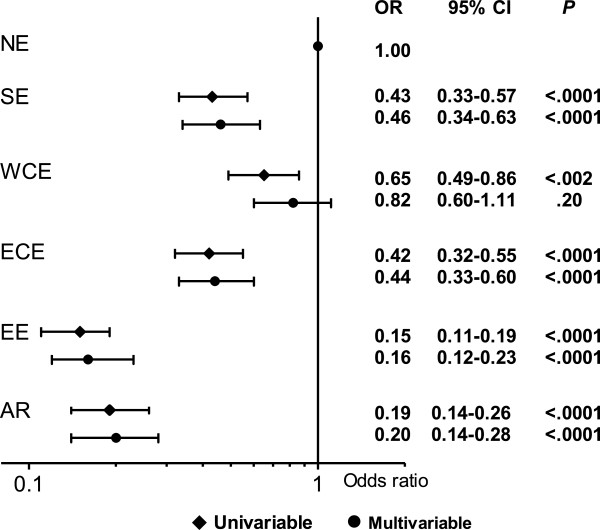
**Odds Ratio (OR) of having suppressed HIV-RNA (<500 copies/ml) during more than 90% of time spent on cART.** Multivariable model was also adjusted for: gender, age, race**,** risk factor for HIV acquisition, hepatitis B/C status, type of cART regimen, being ART naïve at initiation of cART, year of cART initiation, baseline CD4-cell count and HIV-RNA. SE, Southern Europe: Greece, Israel, Italy, Portugal, Spain; WCE, West Central Europe: Austria, Belgium, France, South Germany, Luxembourg, Switzerland; NE, Northern Europe: Denmark, Finland, North Germany, Ireland, the Netherlands, Norway, Sweden, the United Kingdom; ECE, East Central Europe: Bulgaria, Croatia, the Czech Republic, Hungary, Poland, Romania, Serbia and Slovakia; EE, Eastern Europe: Belarus, Estonia, Latvia, Lithuania, the Russian Federation and the Ukraine; AR, Argentina. cART, combination antiretroviral therapy; HIV-RNA, viral load.

Regional differences in the response to cART remained after adjustment for other factors that potentially might influence the proportion of time with suppressed HIV-RNA. Compared with NE, the odds of a suppressed HIV-RNA during at least 90% of the time on cART were approximately 5-6–fold lower in EE and AR, and 2-fold lower in the ECE and SE (Figure 5). Other factors significantly associated with the odds of having suppressed HIV-RNA at least 90% of the time on cART were being ART naïve at initiation of cART vs. ART-experienced [1.43(1.19-1.71), p < 0.0001], being older [1.16 (1.07-1.25) per additional 10 years, p = 0.0004], starting cART more recently [1.13 (1.11-1.16) per year, p < 0.0001] and being infected via homosexual contact vs. IDU [1.49 (1.13-1.96), p = 0.004]. Patients with a higher HIV-RNA at starting cART [0.51 (0.49-0.54) per log10 higher, p < 0.0001] and those who initiated (un)boosted PI- or Abacavir- vs. NNRTI-based regimens [0.67 (0.53-0.84) p = 0.03, and 0.66 (0.439-0.79) p = 0.001, respectively] had significantly lower odds of having suppressed HIV-RNA at least 90% of the time on cART.

The proportion of patients with HIV-RNA results available from assays with a detection level of 50copies/ml varied across regions: 91%, 90%, 80%, 68%, 62% and 27% in AR, NE, WCE, SE, EC and EE, respectively. However, results remained similar when sensitivity analysis of HCI 4 was performed among these patients (N = 3504) (data not shown).

## Discussion

This is the first study that employs a concise set of indicators, based on current treatment guidelines, to assess and compare the delivery of health care in a large and heterogeneous population of HIV patients across countries with highly varying levels of resources. By testing this new set of HCI, the study addresses an intriguing question of quality of HIV care on a regional level rather than the evaluation of clinical status of an individual patient. Further, this set of HCI could be adjusted and used to evaluate the efficacy of treatment programmes for HIV-positive people elsewhere. The evaluation of health systems performance represents an important yet often overlooked instrument for improving the management of HIV infection. The main focus of the current HCIs is clinical management of HIV patients, rather than clinical outcomes, recently published [15]. The results of this previous study demonstrated an increased mortality in EE, particularly from AIDS related events, which is consistent with our findings herein of poorer HCI, particularly proportion of time virologically suppressed, in EE. More specifically, a data-driven identification of the best level of health care utilisation allows for establishing a clinical benchmark for HIV care which, in turn, enables the detection of gaps and facilitates continuous quality improvement of the management of HIV infection.

The assessment of health care quality in general is an essential component of disease management. A benchmark should represent a clinically realistic level of excellence and at the same time exceed mean performance [16]. To evaluate the utilisation of HIV care we chose specific clinical process-of-care indicators measuring adherence to existing guidelines (HCIs 1–3), as well as quality outcome measures, i.e. the ability to achieve the best clinical response after the initiation of cART (HCI 4). We aimed to identify a set of simply measurable HCI, which could be easily implemented in any setting.

Although current analysis documents substantial differences in the utilisation of HIV care across Europe and Argentina, underlying reasons for these differences should be a subject for further investigation. Whereas there were only few and generally minor differences between NE, WCE, and SE in most HCIs, EE, AR and to some extent ECE differed significantly from the rest of the regions. In terms of time of cART initiation, a significantly higher proportion of patients in ECE, EE and AR started cART at a CD4-cell count <200 cells/mm^3^ or after an AIDS diagnosis, even in recent years despite changes to guidelines [1-3]. Nevertheless, it is reassuring to see a slight increase over time in the proportion of patients starting cART at higher CD4-cell counts in EE. This is consistent with the fact that access to cART has substantially increased in this region after 2006 [5]. Late cART initiation could also to some extent be explained by late presentation. In our study most patients in EE starting cART at low CD4-cell count are known HIV-infected, but followed without cART, whereas patients in other regions to a larger extent are late presenters (data not shown). This likely reflects the inclusion criteria for the study. Patients are required to have a pre-booked, outpatient appointment and hence late presenters, who are more likely to die or be admitted to hospital, will not be recruited to the study until they are under routine follow-up.

The use of PCP-chemoprophylaxis was fairly stable over the years in SE, WCE, NE and ECE. Argentina is remarkable by the highest use of PCP-chemoprophylaxis which, for example, could be explained by lower access to cART in the country in the earlier years (<2004), the low cost of co-trimoxazole and a high proportion of late presenters [17]. EE is considered to have a suboptimal access to cART and a high proportion of AIDS patients [5]; however, the level of PCP-chemoprophylaxis was rather low in this region, despite the fact that this is a very cheap and accessible method to prevent the disease.

Substantial differences in the frequency of laboratory monitoring for HIV-disease status were observed across the regions. Current guidelines recommend clinical and laboratory evaluation of HIV-disease status at least every half year before initiation of cART and on a 6–12 month basis in clinically stable patients on cART with suppressed HIV-RNA [1,3]. Adequate monitoring of untreated patients is important in terms of the timely initiation of cART and the prevention of AIDS and, ultimately, death.

When compared to NE, patients treated with cART in all other regions, except WCE, had significantly lower odds of having suppressed plasma HIV-RNA levels for sustained periods of time. This was particularly true for EE and AR. The observed regional differences were over and above what could be explained by differences in patient characteristics and other factors we could adjust for. There might be some factors, such as socio-economic and infrastructure aspects, which play a role and for which we yet do not have data [18]. The majority of patients from EE acquired HIV by IDU. This population group is usually younger, more mobile, less integrated into society and less likely to address health care [19,20]. The domination in mode of HIV transmission of the IDU population and the likely higher level of active IDU among HIV-positive patients in EE might partially explain the inferior outcome in this region [18,20]. However, sensitivity analysis excluding patients with IDU as a HIV risk factor showed consistent results (data not shown).

The main goal of cART is to restore the immune system through the maximal suppression of viral replication and thereby minimise the risk of AIDS developing and death [3]. Regional comparisons of clinical outcomes have recently been published [15]. Instead, the cut-off of 90% of time on cART spent with suppressed HIV-RNA was chosen as a benchmark criteria based on a recent analysis of significantly higher rates of virological failure in patients spending less than 90% of time on cART with suppressed HIV-RNA [14]. While EuroSIDA does not have good data on adherence, likely an important component of any HCI, spending >90% of the time of cART virologically suppressed is probably a marker of good adherence, as those most adherent to cART are most likely to continually suppress viremia [14].

Limitations of the current analysis should be noted. Further research is needed to investigate the underlying reasons for the identified gaps in HIV care. Late cART initiation, not using PCP prophylaxis in patients with CD4-cell count <200 cells/mm^3^ or not measuring CD4-cell count and HIV-RNA can be equally related to the specifics of health care infrastructure as well as to the patients’ behaviour. Addressing the issues above will help to plan targeted interventions and quality improvement programmes: whether it should be health care system-, provider- or patient- oriented. Information on countries’ laws regarding HIV-testing might help to better understand the impact of opt-out HIV-testing on late presentation of HIV-infection. EuroSIDA is currently collecting new data and investigating the underlying reasons for differences in HCIs. A health care questionnaire to address site-specific issues within health care utilisation is currently under development. The main HCI we focused on was the virologic response to cART, and HIV-RNA was measured less frequently in EE compared to the other regions of Europe. Therefore, we may have overestimated the proportion of time virologically suppressed in EE, however this fact was taken into account by censoring the follow-up if there were no HIV-RNA measurement for >6 months. Finally, EuroSIDA includes predominantly major HIV clinics, and the cohort might not be representative for the entire HIV-positive population in Europe, and especially so in EE. Hence the real life situation in terms of HIV health care in EE is likely even worse than presented here.

Comparative analysis of health care utilization and identification of benchmarks and gaps should help to better address the problem of managing HIV infection showing a path towards improvement. Lower-income regions would not necessarily soon reach the same level of health care as in high-income regions, but it is important to continually seek improvement. With the current paper we aim to focus public health attention on the HIV care situation in Eastern Europe, prompting further investigations and interventions as appropriate

## Conclusion

The criteria we have applied in this evaluation of health care utilisation for HIV patients were based on current HIV treatment guidelines and may serve as a tool for benchmarking the clinical management of HIV infection in Europe and elsewhere. These criteria may further be adjusted according to the local setting and, for example, include aspects of hepatitis and/or tuberculosis coinfection. By comparison of HIV care in different regions, the study suggests a data-driven benchmark for HIV care based on the results obtained from the region with the best performance. At the same time, the study emphasizes areas for improvement within those regions. Further research is needed to investigate and better comprehend the underlying reasons for the gaps identified. This study also highlights an important public health message by documenting substantial differences in health care utilisation across regions, and urgent measures should be taken in order to improve access, uptake and response to cART, particularly in Eastern Europe, in line with the global commitment to universal access to prevention, treatment and care.

## Competing interests

The authors declare that they have no competing interests.

## Authors’ contributions

DNP contributed with data interpretation and was responsible for writing the manuscript. JR performed data analysis and contributed in writing manuscript. AM contributed with ideas for data analysis and supervision of data analysis; and contributed in writing manuscript. JVL reviewed the manuscript and contributed to writing it. AGR and IAK contributed with national coordination, data collection, consultations on the HIV-situation in Eastern Europe and writing manuscript. EB contributed with data collection and writing manuscript. JG and ML contributed with national coordination, data collection and writing manuscript. JDL suggested the concept of HIV health care benchmarking, contributed with ideas for data analysis, interpretation of data, and writing the manuscript. OK contributed with ideas for data analysis and interpretation of data, writing the manuscript and overall project supervision. All authors read and approved the final manuscript.

## Funding

Primary support for EuroSIDA is provided by the European Commission BIOMED 1 (CT94-1637), BIOMED 2 (CT97-2713), the 5th Framework (QLK2-2000-00773) and the 6th Framework (LSHP-CT-2006-018632), and the 7th Framework (FP7/2007-2013, EuroCoord n° 260694) programmes. Current support also includes unrestricted grants by Gilead, Pfizer, BMS, Merck and Co. The participation of centres from Switzerland was supported by The Swiss National Science Foundation (Grant 108787).

## Pre-publication history

The pre-publication history for this paper can be accessed here:

http://www.biomedcentral.com/1471-2334/12/229/prepub
